# Case: Unexpected development of severe penicillin allergy and review of literature

**DOI:** 10.1002/ccr3.5248

**Published:** 2022-01-20

**Authors:** Rauno J. Harvima, Ilkka T. Harvima

**Affiliations:** ^1^ Department of Dermatology Kuopio University Hospital and University of Eastern Finland Kuopio Finland

**Keywords:** amoxicillin, benzylpenicillin, cefaclor, penicillin allergy, phenoxymethylpenicillin, specific IgE

## Abstract

A 54‐year‐old man developed a severe anaphylactic penicillin allergy after 16 years and 5 standard erysipelas treatments by intravenous benzylpenicillin and/or oral phenoxymethylpenicillin without any symptoms of allergy. It is recommended to analyze specific IgE antibodies for phenoxymethylpenicillin, benzylpenicillin, amoxicillin, and cefaclor to select an appropriate antibiotic.

## Graphical abstract

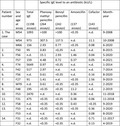

## 1 CASE HISTORY

In September 2008, a 54‐year‐old man (BMI 41.9) was suffering from left knee arthrosis, chronic obstructive pulmonary disease, and venous insufficiency of legs. The history for dermatitis, allergies, and atopy for the patient, 6 siblings, and parents was negative. The patient had a smoking history of 15 cigarettes a day for 37 years. The medication included only inhaled aerosol, Seretide 50 µg/250 µg (salmeterol/fluticasone propionate).

In 1983 and 1996, varicose vein surgeries were performed on the right leg. Seven years later, the patient was treated for an infected right leg wound by using oral cefadroxil.

The patient suffered twice from right leg erysipelas in 1990s, and on both occasions, it was treated with oral phenoxymethylpenicillin. In April 2006, the erysipelas was treated first with intravenous (i.v.) benzylpenicillin, then with i.v. cefuroxime followed by oral clindamycin. A relapse occurred 3 weeks later, and it was treated with i.v. and oral clindamycin. Erysipelas reappeared in October 2006, and it was treated, again, with clindamycin. As the prophylaxis of erysipelas, an oral phenoxymethylpenicillin was started but was soon discontinued because of adverse effects in stomach.

In October 2007, a surgical operation was performed due to varicose vein relapse and ulcer in the right leg, and the patient received one 3‐gram dose of i.v. cefuroxime.

In May 2008, the patient, suffering from the 5th erysipelas, was treated with i.v. clindamycin for 1 day followed by i.v. benzylpenicillin for 3 days. Thereafter, the patient was discharged from the hospital with oral phenoxymethylpenicillin for 3 weeks.

The 6th and severe right leg erysipelas took place 3 months later in early September 2008. In the emergency care unit, the patient was suspected to have an initial sepsis without findings suggestive for necrotizing fasciitis. The patient was planned to be treated with i.v. clindamycin (600 mg × 4 per day) and benzylpenicillin (4 million IU × 4 per day). However, soon after initiation of the drug infusion, that is, within about 30 min, the face became symmetrically swollen, which was considered to be an allergic reaction. The blood pressure decreased down to 96/48 mmHg, the patient received oral cetirizine (10 mg) and i.v. hydrocortisone sodium succinate (125 mg). In the following morning, the patient felt better without fever, and the blood pressure was normalized (124/67 mmHg). Slight facial redness and eyelid edema were seen. However, there was still clear edema and erythema in the right leg. In laboratory analyses, the level of serum C‐reactive protein (CRP) was 198 mg/L and blood leukocyte count was 20.2 × 10^9^/L. Therefore, the treatment with i.v. clindamycin was continued but without benzylpenicillin. The leg edema and erythema disappeared during the following days, like decreased steadily, also the CRP level and leukocyte count down to 16 mg/L and 10.1 × 10^9^/L, respectively. Upon discharge, oral clindamycin capsules were prescribed for 4.5 weeks without adverse effects.

In the follow‐up, the patient was free of symptoms, until the 7th right leg erysipelas occurred in August 2014. The infection was treated successfully with i.v. and oral clindamycin.

In order to investigate the cause of allergic/anaphylactic reaction during the infusion of clindamycin and benzylpenicillin, laboratory analyses were performed. One week after the allergic/anaphylactic reaction serum total IgE level was markedly elevated, as well as serum levels of specific IgE to benzylpenicillin and phenoxymethylpenicillin. Instead, specific IgE to amoxicillin was normal (Table 1).

**TABLE 1:** Total number of assays and cases with elevated specific IgE to an antibiotic

			Specific IgE level to an antibiotic (kU/L)				
Patient number	Sex and age at assay	Total IgE (1198 assays)	Phenoxy methyl penicillin (264 assays)	Benzyl penicillin (242 assays)	Amoxicillin (137 assays)	Cefaclor (143 assays)	Month‐year
1. The case	M54	1093	>100	>100	<0.35	n.d.	9‐2008
	M54	973	307.5	137.5	n.d.	11.1	10‐2008
	M66	156	2.93	0.77	<0.35	0.08	6‐2020
2.	F50	95	0.83	<0,35	n.d.	n.d.	8‐2015
3.	F56	n.d.	15.1	2.93	1.46	0.04	5‐2020
	F57	159	4.48	0.72	0.37	0.05	4‐2021
4.	F74	5649	0.97	<0.35	n.d.	n.d.	1‐2014
5.	F42	517	2.97	n.d.	n.d.	n.d.	7‐2014
6.	F56	n.d.	0.61	<0.35	n.d.	0.16	8‐2020
7.	F27	91	1.41	n.d.	<0.35	2.56	9‐2019
8.	M70	n.d.	0.61	<0.35	<0.35	n.d.	2‐2020
9.	F48	195	<0.35	<0.35	11.2	n.d.	2‐2019
10.	F53	2470	n.d.	n.d.	0.36	n.d.	11‐2018
11.	F31	475	<0.35	<0.35	0.38	0.06	11‐2018
12.	F58	n.d.	<0.35	<0.35	0.43	<0.35	9‐2019
13.	F53	n.d.	<0.35	<0.35	<0.35	0.36	3‐2020
	F53	n.d.	n.d.	n.d.	n.d.	0.08	8‐2020
14.	F31	n.d.	<0.35	<0.35	n.d.	0.71	11‐2017
	F32	n.d.	<0.35	<0.35	n.d.	0.13	4‐2019

Note

The decision for assays is based on the clinician's evaluation upon examination of a patient. M = male, F = female, “n.d.” denotes not determined. Total number of IgE assays represents all atopy studies.

Normal reference value for total IgE is 0–100 kU/L. For antibiotic specific IgE value for normal or negative reference value for all is <0.35 kU/L.

The results represent the population of 251,000 in the Kuopio University Hospital District in Northern Savo province during January 2010 to August 2021.

One month later, serum total IgE was decreased by about 10%. By using a dilution method, a more accurate estimation for the specific IgE was obtained: for benzylpenicillin it was 137.5 kU/L, and for phenoxymethylpenicillin very high 307.5 kU/l. Specific IgE to cefaclor was also elevated, 11.1 kU/L. Blood eosinophilic leukocytes were marginally elevated (0.5 × 10^9^/L, ref. 0.1–0.4 × 10^9^/L). Later the patient's eosinophil counts were measured 6 times being within normal range.

In June 2020, additional laboratory analyses were performed. Serum total IgE was decreased down to 156 kU/L. Also, specific IgE levels to benzylpenicillin (0.77 kU/L) and phenoxymethylpenicillin (2.93 kU/L) were markedly decreased. Specific IgE antibodies to amoxicillin and cefaclor were normal.

In order to obtain information for comparison and on the prevalence of elevated specific IgE to these antibiotics, a search for laboratory values in the hospital district of Kuopio University Hospital (population about 251,000) between January 2010 and August 2021 was performed. The results of 13 other patients are shown in Table 1. As a summary, clinically relevant increases (>0.8 kU/L) in specific IgE to phenoxymethylpenicillin were detected only in 5 other patients.

Only two patients had elevated specific IgE levels simultaneously to both phenoxymethylpenicillin and amoxicillin. There was one patient with either of these and two patients with simultaneous specific IgE to phenoxymethylpenicillin and cefaclor. One patient showed markedly elevated specific IgE to amoxicillin (11.2 kU/L) without any increase in IgE to phenoxymethylpenicillin and benzylpenicillin.

## 2 DISCUSSION

According to the literature, an anaphylactic reaction to penicillin is very rare, about 0.001% in parenteral and 0.0005% in tablet dosing.^1^ In the Western countries, about 10% of adults declare to be allergic for penicillin, and adverse effects, like stomach symptoms, are believed to mean allergy. However, the real penicillin allergy is much more rare than reported: of patients claiming to be allergic for penicillin, up to 99% of them still tolerate penicillin.^2^ When making estimations about real penicillin allergy, it is of importance to document very carefully the facts to the patient file for future evaluation.

The allergic/anaphylactic reaction in the present case was very likely due to i.v. infusion of benzylpenicillin, because of the very high level of specific IgE to benzylpenicillin (137.5 kU/L) and the reaction subsided when continuing with clindamycin alone. Interestingly, this patient had been treated with the same i.v. benzylpenicillin followed by oral phenoxymethylpenicillin about 2–3 months earlier without any adverse effects. Therefore, it is assumed that the patient was sensitized at the end of the oral treatment with phenoxymethylpenicillin. It is, however, possible that the septic‐like erysipelas may have had some additional effect on the clinical outcome. Of note is the observation, that the sensitization with severe clinical reaction can take place unexpectedly even after a heavy and long background of penicillin use for erysipelas.

In 2008, serum tryptase analysis was not available in this hospital. This assay might have given additional information about mast cell activation. A positive skin prick‐test and slightly elevated specific IgE levels are not reliable to predict IgE‐dependent penicillin allergy.

An interesting finding was that the specific IgE to amoxicillin was normal, even though specific IgE levels to benzylpenicillin and phenoxymethylpenicillin were very high. In the case of amoxicillin, a direct oral exposition can be performed if a mild delayed‐type reaction has occurred. In the literature, there are conflicting views on the direct exposition with amoxicillin, if a patient has experienced an immediate‐type reaction. Therefore, it is not recommended to be used, if an IgE‐mediated reaction has taken place from another penicillin type. However, a direct amoxicillin exposition is considered to be safe, if more than 10 years have gone since the reaction.^3^ However, in an Australian multicenter study,^4^ if penicillin‐associated rash was over 1 year earlier, a direct challenge was considered safe for low‐risk patients, but skin testing for high‐risk patients. According to present findings on the case and other cases with positive IgE to antibiotics, amoxicillin exposition may not be reliable in showing immediate allergies for phenoxymethylpenicillin or benzylpenicillin. Therefore, the exposition should be done with penicillins. However, due to the severity of the reaction, it was not performed in this case.

In the present case, almost 12 years later, serum total IgE level was decreased by about 84%. This is in accordance with previously published data: IgE‐mediated reactions to beta‐lactams have decreased by 80% in 10 years as determined by skin prick tests.^1^ Also, the levels of specific IgE of our patient to both benzylpenicillin and phenoxymethylpenicillin were decreased by 99% during about the same time, and for cefaclor, no specific IgE was detected.

The levels of specific IgE to cefaclor were elevated in this case, as also to benzylpenicillin and phenoxymethylpenicillin. The patient had been treated 5 years earlier with an oral 1st generation cephalosporin, cefadroxil. Also, the patient had been treated about 2 years earlier with some i.v. doses and almost one year earlier with a single i.v. dose of 2nd generation drug, that is, cefuroxime, before surgical operation. Cefaclor is, like cefuroxime, a 2nd generation cephalosporin. Since the cross‐reaction between cefuroxime and penicillin is very low,^2^ it is likely that sensitization to cefaclor had developed by previous i.v. cefuroxime doses.

Although the levels of specific IgE to both penicillins and cefaclor in our patient have markedly decreased or even disappeared, it is recommended to avoid beta‐lactam antibiotics and also 2nd generation cephalosporins—until exposition has been performed.

Amoxicillin can cross‐react, for example, with cefaclor, cefadroxil, and cefalexin. The ratio between cefadroxil and amoxicillin is 12%, but higher percentages of even 27% has also been reported^5^

The cross‐reactions of beta‐lactam antibiotics can result from several structures in the molecule: thiazolidine ring (penicillin), dihydrothiazine ring (cephalosporins), R1 and R2 side chains, or several positions at the same time, but mostly due to side chains.^1^ As a conclusion, it is very difficult to predict in advance the possible cross‐reaction based on the drug itself or on the drug group.

In the literature, a cross‐reaction between penicillin and cephalosporins has been reported to be at the level of 10%, but thereafter it has been found that the early cephalosporin preparations contained penicillin as contamination, leading to over‐estimation of the cross‐reactivity; the present estimate for this cross‐reactivity is 1%–2.5%.^1^, ^5^, ^6^ The cross‐reaction between penicillin and carbapenems is less than 1%. Monobactams do not cross‐react with neither penicillin nor carbapenems.^1^

In the clinical practice, physicians may face the situation that penicillin is the most recommended drug. In such a case, desensitization therapy, first with phenoxymethylpenicillin and later with benzylpenicillin, might be possible to perform, but it requires several weeks to complete the procedure.^7^

In this report, only 2 men and 12 women, tested positive for elevated specific IgE to an antibiotic, were found in about 12 years. The female predominance over males is in concordance with a recent review that a drug allergy is more frequently reported in adult females than males, and 57.9%–73.9% of the drug‐induced anaphylaxis cases were among females.^8^

The search for elevated specific IgE to antibiotics in this hospital district revealed only 5 relevant positive results for penicillin in over 11 years, being in accordance with the estimation elsewhere. However, some rare patients can unexpectedly develop a severe allergy even after a long history of penicillin use, like did the present patient. Also, although he had extremely high levels of IgE antibodies against phenoxymethylpenicillin, benzylpenicillin, and cefaclor, no IgE antibodies were detected against amoxicillin. Therefore, every time an immediate reaction to beta‐lactams develops, it is recommended to analyze specific IgE to penicillins, amoxicillin, and cephalosporin, as well as total IgE, in order to obtain more accurate estimations for allergy and cross‐reactions. This wide spectrum of the allergy potential of penicillin variants and cephalosporins will be of importance for example in oral surgery of bone grafts that an adequate antibiotic is available to reduce the risk of complications.^9^

## CONFLICT OF INTEREST

Authors do not have any relevant conflict of interests.

## AUTHOR CONTRIBUTIONS

Both authors have equally participated in the preparation of this article.

## ETHICAL APPROVAL

Kuopio University Hospital with Ethics Committee has given permit to publish this patient case and the background allergy laboratory data as reference.

## CONSENT

The patient has given a written consent to publish this patient case.
